# Association of Preexisting Interstitial Lung Abnormalities With Immune Checkpoint Inhibitor–Induced Interstitial Lung Disease Among Patients With Nonlung Cancers

**DOI:** 10.1001/jamanetworkopen.2020.22906

**Published:** 2020-11-12

**Authors:** Kiyofumi Shimoji, Takeshi Masuda, Kakuhiro Yamaguchi, Shinjiro Sakamoto, Yasushi Horimasu, Taku Nakashima, Shintaro Miyamoto, Hiroshi Iwamoto, Kazunori Fujitaka, Hironobu Hamada, Sachio Takeno, Michihiro Hide, Jun Teishima, Hideki Ohdan, Noboru Hattori

**Affiliations:** 1 Department of Respiratory Internal Medicine, Hiroshima University Hospital, Minami-ku, Hiroshima, Japan; 2 Department of and Otolaryngology and Head and Neck Surgery, Hiroshima University Hospital, Minami-ku, Hiroshima, Japan; 3 Department of Dermatology, Hiroshima University Hospital, Minami-ku, Hiroshima, Japan; 4 Department of Urology, Hiroshima University Hospital, Minami-ku, Hiroshima, Japan; 5 Department of Gastroenterological and Transplant Surgery, Hiroshima University Hospital, Minami-ku, Hiroshima, Japan

## Abstract

**Importance:**

Immune checkpoint inhibitor–induced interstitial lung disease (ICI-ILD) is clinically serious and life-threatening. Preexisting interstitial lung abnormalities have been shown to be risk factors for ICI-ILD in patients with lung cancer.

**Objective:**

To evaluate whether interstitial lung abnormalities are associated with ICI-ILD in patients with nonlung cancers.

**Design, Setting, and Participants:**

This cohort study was conducted between December 2015 and May 2019 at Hiroshima University Hospital. A total of 199 consecutive patients with head and neck cancer, malignant melanoma, oral cavity cancer, urological cancer, and gastrointestinal cancer who received anti–programmed cell death 1 (PD-1) antibody monotherapy were included. Data analysis was conducted from December 2015 to May 2019.

**Main Outcomes and Measures:**

The associations between potential risk factors and the development of ICI-ILD were examined. Information on patient characteristics before antibody administration, including chest computed tomography findings, was obtained. The diagnosis of ICI-ILD was defined as abnormal computed tomography shadows occurring during treatment with anti-PD-1 antibodies.

**Results:**

A total of 199 patients were enrolled in the study. The median (range) age was 66 (20-93) years, and most patients (133 [66.8%]) were men. Nineteen patients (9.5%) developed ICI-ILD. There was no significant difference in the baseline characteristics between patients with and without ICI-ILD. The logistic regression analyses revealed that interstitial lung abnormalities were associated with increased risk of ICI-ILD (odds ratio, 6.29; 95% CI, 2.34-16.92; *P* < .001), and ground glass attenuation in interstitial lung abnormalities was an independently associated risk factor (odds ratio, 4.05; 95% CI, 1.29-12.71; *P* = .01).

**Conclusions and Relevance:**

In this cohort study, preexisting interstitial lung abnormalities, including ground glass attenuation, were risk factors associated with ICI-ILD in patients with nonlung cancers. This observation is consistent with previously reported findings in patients with lung cancer. Therefore, we should pay more attention to the development of ICI-ILD in patients with interstitial lung abnormalities, regardless of cancer type.

## Key Points

**Question:**

Are interstitial lung abnormalities, which are minor interstitial shadows on lung computed tomography, associated with immune checkpoint inhibitor induced–interstitial lung disease in patients with nonlung cancers?

**Findings:**

In this cohort study of 199 patients with advanced nonlung cancers treated with anti–programmed cell death 1 antibody monotherapy, patients with preexisting interstitial lung abnormalities had significantly increased risk of immune checkpoint inhibitor induced–interstitial lung disease.

**Meaning:**

These findings suggest that greater attention should be accorded to the development of immune checkpoint inhibitor induced–interstitial lung disease in patients with nonlung cancers and interstitial lung abnormalities.

## Teaser

This cohort study evaluates whether interstitial lung abnormalities are associated with immune checkpoint inhibitor–induced interstitial lung disease in patients with nonlung cancers.

## Introduction

Anti–programmed cell death 1 (PD-1) antibodies, an immune checkpoint inhibitor (ICI), exert antitumor effects by suppressing the immune tolerance to cancer cells. Recent clinical trials have demonstrated such antitumor effects, and these antibodies are approved for use for the treatment of several advanced cancers.^1,2,3,4,5^ However, ICI-related adverse events are observed in patients receiving anti–PD-1 antibodies. Among them, ICI-induced interstitial lung disease (ICI-ILD) is a clinically serious and life-threatening toxic effect.^6,7^ The reported prevalence of ICI-ILD is 4.1% in patients with lung cancer, 4.1% in patients with kidney cell carcinoma, and 1.6% in patients with malignant melanoma.^8^ Therefore, it is important to determine the risk factors for ICI-ILD to pay more attention to the development of ICI-ILD and initiate prompt treatment in patients with risk factors.

Preexisting ILD has been reported as a risk factor for the development of ICI-ILD in patients with non–small cell lung carcinoma (NSCLC).^9,10^ In addition, we previously reported that interstitial lung abnormalities (ILAs) are a risk factor for ICI-ILD in patients with NSCLC.^11^ ILAs are generated by aging or smoking,^12,13,14^ among other factors, and minor interstitial shadows on lung computed tomography (CT) in individuals with no known ILD are present in 14% of treatment-naive patients with NSCLC.^15^ However, ILAs in patients with nonlung cancers would not be recognized before the administration of anti-PD-1 antibodies by physicians. In addition, there have been no reports on the risk factors for ICI-ILD in patients with nonlung cancers. Therefore, we aimed to investigate whether any patient characteristics, including ILAs, were associated with increased risk of ICI-ILD in patients with nonlung cancers.

## Methods

### Study Design and Participants

Consecutive patients with head and neck cancer, malignant melanoma, oral cavity cancer, urological cancer, gastrointestinal cancer, or malignant lymphoma (diagnosed according to *International Statistical Classification of Diseases and Related Health Problems, Tenth Revision*) who received anti-PD-1 antibodies (nivolumab or pembrolizumab) at Hiroshima University Hospital between December 2015 and May 2019 were retrospectively enrolled. Information on patient characteristics before anti-PD-1 antibody administration, including chest CT findings, was obtained. Tumor responses were evaluated using the Response Evaluation Criteria for Solid Tumors version 1.1. To obtain patient consent, this study applied the opt-out method. We posted information about this study on the home page of our institution and guaranteed that the enrolled patients had an opportunity to refuse to participate in this study. In addition, we clarified the period for accepting the intention of refusal. This study was approved by the Hiroshima University institutional review board and conducted in accordance with the ethical standards established by the Declaration of Helsinki.^16^ This report followed the Strengthening the Reporting of Observational Studies in Epidemiology (STROBE) reporting guideline.

### Evaluation of CT Findings

CT data were obtained at the end-inspiration phase in the supine position using a CT scanner. We investigated the presence of abnormal findings, including preexisting ILAs on CT. The presence of ILAs was determined by observations such as ground glass attenuation (GGA), reticulation, honeycombing or traction bronchiectasis, diffuse centrilobular nodularity, and nonemphysematous cysts on CT images^12,13^ within 6 months before anti-PD-1 antibody administration (Figure). These findings were classified using previously reported criteria.^12,17^ The extent of ILAs was defined as focal or unilateral, patchy (<5% of the lung).^12,17^ Using a sequential reading method, CT images were evaluated by 3 pulmonologists (K.S., T.M., and S.S.) who were unaware of other clinical findings or patient outcomes.^12,17^ In brief, reader 1 evaluated all CT images, and reader 2 reviewed ILA images and 20% of the non-ILA images that were evaluated by reader 1. Images with discordant evaluations were further reviewed by reader 3, and final decisions were agreed by consensus among the 3 readers. The correlation between reader 1 and reader 2 was determined by evaluating the Spearman rank correlation coefficient.

**Figure.**
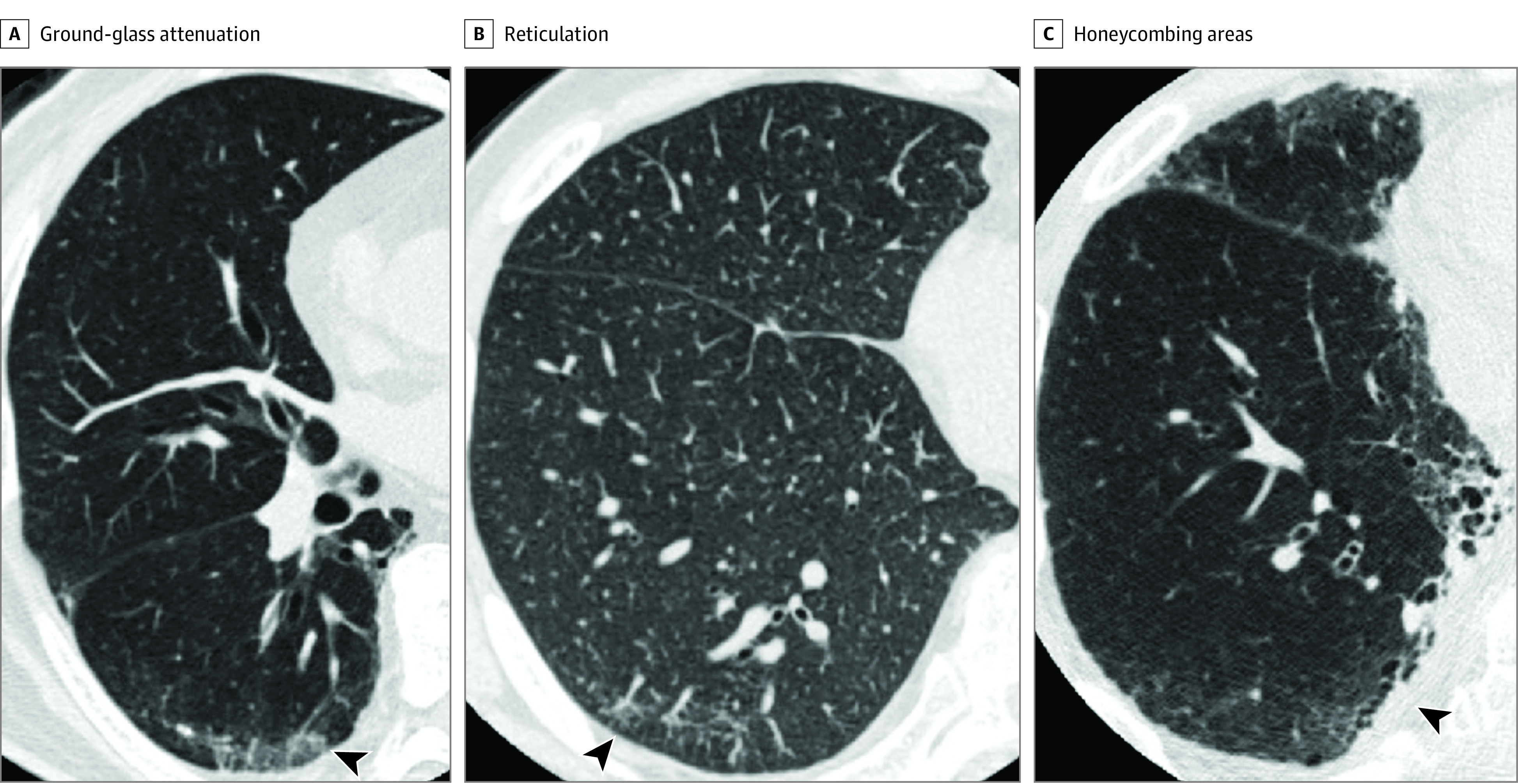
Computed Tomography Images of Interstitial Lung Abnormalities

### Diagnosis of ICI-ILD

The diagnosis of ICI-ILD was defined as follows: (1) new abnormal shadows occurring on chest CT during treatment with anti-PD-1 antibodies (eFigure 1 in the Supplement), (2) exclusion of pulmonary infection (ie, pneumonia that did not improve even after antibiotic administration or no bacteria in the sputum culture), (3) exclusion of heart failure using laboratory data and/or transthoracic echocardiography, and (4) exclusion of tumor progression using laboratory data and version 1.1 of the Response Evaluation Criteria in Solid Tumors. In each case, the radiographic patterns of the pneumonitis were classified according to the American Thoracic Society/European Respiratory Society international multidisciplinary classification of interstitial pneumonias, as described previously^18^: (1) usual interstitial pneumonia pattern, (2) nonspecific interstitial pneumonia (NSIP) pattern, (3) organizing pneumonia (OP) pattern, (4) diffuse alveolar damage (DAD) pattern, or (5) not applicable. This classification was evaluated by 2 pulmonologists (K.S. and T.M.), who did not have information on the presence or absence of ILA or characteristics for each patient. Final decisions were agreed on by consensus between both pulmonologists. The time frame for chest CT was determined by clinicians based on the patient’s symptoms, blood tests, and chest radiographic findings. ILD was evaluated based on the National Cancer Institute Common Terminology Criteria for Adverse Events, version 5.0.

### Statistical Analysis

Results are expressed as medians (range) or means (SD). Comparisons between 2 groups were performed using Pearson χ^2^ or the Wilcoxon rank sum test. Univariate and multivariate analyses using the logistic regression model were used to identify risk factors associated with ICI-ILD. All reported *P* values are 2-sided, and *P* < .05 was considered statistically significant. All statistical analyses were performed using JMP Pro version 14 (SAS Institute).

## Results

### Patient Characteristics

A total of 199 patients were enrolled in the study. The clinical characteristics of these patients are provided in Table 1, and the proportion of carcinomas is indicated in eFigure 2 in the Supplement. The median age was 66 years (range, 20-93 years). Many patients (98 [49.2%]) currently or formerly smoked. Male patients (133 [66.8%]) and patients with Eastern Cooperative Oncology Group performance statuses of 0 or 1 (152 [76.5%]) were predominant. All enrolled patients underwent chest CT. The prevalence of ILAs was 18.5% (37 patients). In the original clinical radiology report, the proportion of patients with ILA was only 27.0% (10 patients). A significant correlation between CT readers in the evaluation of ILA was observed (eTable 1 in the Supplement). In addition, we compared patient characteristics between the groups with and without ILA; the group with ILA was significantly older than the group without ILA (eTable 2 in the Supplement). Nivolumab and pembrolizumab were administered to 177 patients (88.9%) and 22 patients (11.1%), respectively. A total of 34 patients (17.1%) received ICIs at the first line of treatment. The tumor response rate was 32 (16.1%). Among patient characteristics, we noted missing data on smoking history (15 patients [7.5%]) and best tumor response (28 cases [14.1%]). The clinical characteristics of patients with each type of cancer are presented in eTable 3 in the Supplement.

**Table 1.** Patient and CT Characteristics

Characteristic	Patients, No. (%) (N = 199)
Age, median (range), y	66 (20-93)
Sex	
Men	133 (66.8)
Women	66 (33.2)
Smoking status	
Never	86 (43.2)
Current or former	98 (49.2)
Unknown	15 (7.5)
ECOG PS score	
0–1	152 (76.4)
≥2	47 (23.6)
CT characteristics	
Existing abnormal finding	165 (82.9)
Details of abnormal finding	
Preexisting interstitial lung abnormalities	37 (18.6)
Ground glass attenuation	24 (12.1)
Reticulation	20 (10.1)
Honeycombing	5 (2.5)
Traction bronchiectasis	0
Emphysema	46 (23.1)
Lung metastasis	87 (43.7)
Radiation pneumonitis	6 (3.0)
Consolidation	8 (4.0)
Lymphangiosis carcinomatosa	3 (1.5)
Treatment and tumor response	
ICI	
Nivolumab	177 (88.9)
Pembrolizumab	22 (11.1)
Line of ICI therapy	
1	34 (17.1)
2	68 (34.2)
3	52 (26.1)
≥4	45 (22.6)
ICI cycles, No.	
1	14 (7.0)
2	20 (10.1)
3	15 (7.5)
4	24 (12.1)
≥5	126 (63.3)
Best tumor response	
CR	2 (1.0)
PR	30 (15.1)
SD	55 (27.6)
PD	84 (42.2)
Unknown	28 (14.1)

Abbreviations: CR, complete response; CT, computed tomography; ECOG PS, Eastern Cooperative Oncology Group performance status; ICI, immune checkpoint inhibitor; PD, progressive disease; PR, partial response; SD, stable disease.

### Characteristics of ICI-ILD

Of 199 patients, 19 (9.5%) developed ICI-ILD (Table 2). Among the 34 patients (17.1%) who received ICIs as first-line treatment, 4 (11.7%) developed ICI-ILD. Of 68 patients (34.2%) who received ICIs as second-line treatment, 5 (7.4%) developed ICI-ILD. Of the 52 patients (26.1%) who received third-line ICI therapy, 7 (13.5%) developed ICI-ILD. Fourth-line or later ICI therapies were administered to 45 patients (22.6%), of whom 3 (6.7%) developed ICI-ILD. The median time to ICI-ILD development was 91 days (range, 1-350 days). The radiographic patterns of ICI-ILD were OP (6 patients [31.6%]), NSIP (11 patients [57.9%]), and DAD (2 patients [10.5%]). Grade 1 was observed in 15 patients (78.9%), grade 2 in 2 (10.5%), grade 3 in 1 (5.3%), and grade 5 in 1 (5.3%). Steroid therapy was administered to the 4 patients with grade 2 or higher ICI-ILD. Nevertheless, 1 patient died. In the ICI-ILD group, no patients exhibited a complete response, 6 (31.6%) exhibited a partial response, 2 (10.5%) exhibited disease stability, and 8 (42.1%) exhibited disease progression. The proportion of patients with ICI-ILD among those with each type of nonlung cancer was 11.1% (5 of 45 patients) with head and neck cancer, 4.7% (2 of 42 patients) with malignant melanoma, 9.5% (4 of 42 patients) with urological cancer, 12.5% (4 of 28 patients) with oral cavity cancer, and 10.7% (3 of 28 patients) with gastrointestinal cancer (eTable 4 in the Supplement). Tumor progression was excluded in all patients with ICI-ILD. Of 19 patients, 5 (26.3%) had fever and sputum and presented with cough. All 5 patients received antibiotics, and 2 (40.0%) underwent the sputum culture test. In addition, chest radiograph and CT revealed heart failure in 3 of 19 patients (15.8%). Thus, these patients underwent laboratory tests and/or transthoracic echocardiography.

**Table 2.** Characteristics of Patients With Immune Checkpoint Inhibitor-Induced Interstitial Lung Disease

Characteristic	Patients, No. (%) (n = 19)
Line of ICI therapy	
1	4 (21.1)
2	5 (26.3)
3	7 (36.8)
≥4	3 (15.8)
Cycles of ICI, No.	
1	2 (10.5)
2	3 (15.8)
3	1 (5.3)
4	2 (10.5)
≥5	11 (57.9)
ICI	
Nivolumab	16 (84.2)
Pembrolizumab	3 (15.8)
Time to onset of ICI-ILD, median (range), d	91 (1-350)
ICI-ILD pattern	
OP	6 (31.6)
NSIP	11 (57.9)
DAD	2 (10.5)
CTCAE ICI-ILD grade	
1	15 (78.9)
2	2 (10.5)
3	1 (5.3)
4	0
5	1 (5.3)
Steroid use	4 (21.1)
Best tumor response	
CR	0
PR	6 (31.6)
SD	2 (10.5)
PD	8 (42.1)
Unknown	3 (15.8)

Abbreviations: CR, complete response; CTCAE, Common Terminology Criteria for Adverse Events version 5.0; DAD, diffuse alveolar damage pattern; ICI, immune checkpoint inhibitor; ICI-ILD, immune checkpoint inhibitor–induced interstitial lung disease; NSIP, nonspecific interstitial pneumonia pattern; OP, organizing pneumonia pattern; PD, progressive disease; PR, partial response; SD, stable disease.

### Comparison of Characteristics Between Patients With and Without ICI-ILD

The baseline characteristics of patients with and without ICI-ILD did not differ significantly (Table 3; eTable 5 in the Supplement). The proportion of patients with partial responses or complete responses was significantly higher among patients with ICI-ILD than in those without ICI-ILD (6 of 10 [60.0%] vs 25 of 130 [19.2%]; *P* = .03). In contrast, the proportion of patients with ILAs was significantly higher among patients with ICI-ILD than among those without (10 of 19 [52.6%] vs 27 of 153 [15.0%]; *P* < .001) (Table 3). Furthermore, the proportions of patients with GGA and reticulations were higher among patients with ICI-ILD than among those without (GGA: 7 of 19 [36.8%] vs 18 of 180 [10.0%]; *P* < .001; reticulation: 5 of 19 [26.3%] vs 15 of 180 [8.3%]; *P* = .01). However, the proportions of patients with abnormal shadows, including emphysema, metastatic lung tumors, and radiation pneumonitis, did not differ significantly between the 2 groups. In urological or gastrointestinal cancers, the proportion of patients with ILAs was significantly higher in patients with ICI-ILD than those without, although the number of patients was small (eTable 6 in the Supplement).

**Table 3.** Comparison of Characteristics and CT Findings Between Patients With and Without ICI-ILD

Characteristic	Patients, No. (%)		*P* value
	With ICI-ILD (n = 19)	Without ICI-ILD (n = 180)	
Age, median (range)	71 (20-80)	65 (20-93)	.43
Sex			
Men	14 (73.7)	119 (66.1)	.50
Women	5 (26.3)	61 (33.9)	
Smoking status			
Never	6 (31.6)	92 (51.1)	.18
Current or Former	10 (52.6)	76 (42.2)	
ECOG PS			
0–1	13 (68.4)	139 (77.2)	.39
≥2	6 (31.6)	41 (22.8)	
Line of ICI therapy			
1	4 (21.1)	30 (16.7)	.62
≥2	15 (78.9)	150 (83.3)	
CT characteristics			
Existing abnormal finding	17 (89.5)	148 (82.2)	.42
Preexisting interstitial lung abnormalities	10 (52.6)	27 (15.0)	
Type of interstitial lung abnormalities			
Ground glass attenuation	7 (36.8)	18 (10.0)	<.001
Reticulation	5 (26.3)	15 (8.3)	.01
Honeycombing	0	5 (2.8)	.46
Traction bronchiectasis	0	0	NA
Emphysema	2 (10.5)	44 (24.4)	.17
Lung metastasis	5 (26.3)	82 (45.6)	.10
Radiation pneumonitis	1 (5.3)	5 (2.8)	.54
Consolidation	0	8 (4.4)	.34
Lymphangiosis carcinomatosa	0	3 (1.7)	.57

Abbreviations: CT, computed tomography; ECOG PS, Eastern Cooperative Oncology Group Performance Status; ICI-ILD, immune checkpoint inhibitor–induced interstitial lung disease; NA, not applicable.

### Logistic Regression Analyses of the Risk Factors Associated With ICI-ILD

Preexisting ILAs (odds ratio [OR], 6.29; 95% CI, 2.34-16.92; *P* < .001), GGA (OR, 5.24; 95% CI, 1.83-15.02; *P* = .01), and reticulation (OR, 3.92; 95% CI, 1.24-12.40; *P* = .02) were identified as significant risk factors associated with ICI-ILD in the univariate analysis (Table 4). Considering adjustment factors, we created the following 2 regression models in multivariate analysis. In model 1, we first chose ILA, which was significant in the univariate logistic regression analysis. In addition, we selected age, sex, and smoking history as covariates, which have been reported to be associated with ILA in previous studies.^12,13,14^ This model showed ILA as an independent risk factor for ICI-ILD (OR, 6.42; 95% CI, 1.96-21.03; *P* = .01) (Table 4). In model 2, to identify which type of ILA was an independent risk factor for ICI-ILD development, we chose GGA and reticulation as factors in multivariate analysis because they were significant in the univariate analysis. This model identified GGA (OR, 4.05; 95% CI, 1.29-12.71; *P* = .01) in ILAs to be an independent risk factor associated with ICI-ILD.

**Table 4.** Univariate and Multivariate Analysis of Risk Factors Associated With ICI-ILD

Characteristic	Odds ratio (95% Cl)	*P* value
**Univariate analysis**		
Age, continuous	0.98 (0.95-1.02)	.43
Sex, men vs women	1.43 (0.49-4.17)	.50
Smoking status, never vs current or former	0.49 (0.17-1.42)	.19
ECOG PS, 0-1 vs ≥2	1.56 (0.55-4.37)	.39
Line of ICI therapy, 1 vs ≥2	1.33 (0.41-4.29)	.53
CT characteristics		
Existing abnormal finding	1.83 (0.40-8.35)	.43
Preexisting interstitial lung abnormalities	6.29 (2.34-16.92)	<.001
Type of interstitial lung abnormalities		
Ground glass attenuation	5.24 (1.83-15.02)	.01
Reticulation	3.92 (1.24-12.40)	.02
Emphysema	0.36 (0.08-1.63)	.18
Lung metastasis	0.42 (0.14-1.23)	.11
Radiation pneumonitis	1.94 (0.21-17.56)	.55
**Multivariate analysis**		
Model 1		
Age, continuous	1.25 (0.08-18.80)	.86
Sex, men vs women	1.80 (0.50-6.42)	.36
Smoking status, never vs current or former	0.32 (0.09-1.10)	.07
Preexisting interstitial lung abnormalities	6.42 (1.96-21.03)	.002
Model 2		
Ground glass attenuation	4.05 (1.29-12.71)	.01
Reticulation	2.28 (0.63-8.21)	.20

Abbreviations: CT, computed tomography; ECOG PS, Eastern Cooperative Oncology Group performance status; ICI-ILD, immune checkpoint inhibitor–induced interstitial lung disease.

## Discussion

This study found that preexisting ILAs were risk factors associated with ICI-ILD in patients with nonlung cancers, and GGA in ILAs was an independent risk factor. ICI-ILD is a life-threatening toxic effect, and it was observed in several clinical trials of nonlung cancers.^7,19,20^ However, to our knowledge, risk factors for ICI-ILD in nonlung cancers have not been previously investigated. Therefore, to our knowledge, this is the first study to identify preexisting ILAs as risk factors associated with ICI-ILD in nonlung cancers. We previously reported that preexisting ILAs are risk factors for ICI-ILD in patients with NSCLC patients.^11^ Therefore, greater attention should be paid to the development of ICI-ILD in patients with ILAs regardless of cancer type.

ILAs as risk factors for ICI-ILD depends on the pathological feature of the ILAs. The present study revealed that reticulation and GGA but not honeycombing or traction bronchiectasis were significant risk factors in the univariate analysis, and only GGA was independently associated with ICI-ILD. Reticulation pathologically reflects slight fibrosis and inflammation by lymphocytes in the interstitium of the lung. GGA also involves infiltration of lymphocytes into the interstitium, reflecting active inflammation. However, honeycombing and traction bronchiectasis reflect the destruction of lung alveolar structures due to fibrosis.^8,21^ ICIs exert antitumor effects via the promotion of lymphocyte activity. This could explain why the presence of ILAs, including reticulation and GGA, was associated with ICI-ILD in this study.

It has been reported that ILAs are generated by aging and smoking.^12,13,14^ The definition of ILAs varies, and there are narrow and broad definitions. In a narrow definition, ILA is referred to as dirty lung or preclinical ILD and refers to early or minor ILD in undiagnosed cases. In contrast, ILAs in the broad definition cover early to advanced ILD.^12,15,17^ In this study, minimal interstitial change was narrowly defined as ILA, referring to previous reports.^12,15,17^ The significance of narrowly defined ILAs has been recently reported. Several studies revealed that ILAs are associated with a future decline in respiratory function, the development of interstitial changes,^22^ or the occurrence of lung cancer.^23^

Previous systematic reviews have found that the incidence of ICI-ILD was 4.1% in patients with NSCLC, 1.6% in patients with malignant melanoma, 4.1% in patients with kidney renal cell carcinoma, and approximately 1% to 4% in patients with other cancers.^1,2,3,4,5^ The incidence of ICI-ILD in this study was higher than those previously reported. We believe that this may be attributable to the fact that the number of patients enrolled in this study was lower than that in clinical trials, and the participants in this study were all Japanese, who are reported to have a higher incidence of drug-induced ILD than White patients and patients from other Asian countries.^24^

In this study, ICI-ILD was observed in various lines of ICI, and this result is consistent with those of previous studies.^7^ The duration from the initiation of ICI to the development of ICI-ILD varied considerably, with a median of 87.5 days (range, 1-350 days), which tended to be longer in patients with nonlung cancers than those with NSCLC, according to a previous study. These results suggest that attention should be paid to the development of ICI-ILD regardless of the line and duration of anti-PD-1 antibody administration.

### Limitations

This study has limitations. First, it was retrospective and performed at a single institution. Therefore, a prospective multicenter study is warranted to verify our findings. Second, ICI-ILD was not pathologically diagnosed in this study. Therefore, the ICI-ILD findings on CT could reflect non-ICI-ILD.

## Conclusions

In this cohort study, preexisting ILAs, including GGA, were significant risk factors associated with ICI-ILD in patients with nonlung cancer. This observation is consistent with those previously reported in patients with NSCLC. Therefore, we should focus greater attention on the development of ICI-ILD in patients with ILAs, regardless of the cancer type.

## Notes

Supplement. **eTable 1. **Spearman Rank Correlation Coefficient in the CT Characteristics Among CT Readers **eTable 2. **Comparison of Characteristics Between Patients With and Without Interstitial Lung Abnormalities **eTable 3. **Patient and CT Characteristics **eTable 4. **Proportion of Patients With Immune Checkpoint Inhibitor–Induced Interstitial Lung Disease **eTable 5. **Comparison of Characteristics Between Patients With and Without Immune Checkpoint Inhibitor–Induced Interstitial Lung Disease **eTable 6. **Comparison of CT Findings Between Patients With and Without Immune Checkpoint Inhibitor–Induced Interstitial Lung Disease **eFigure 1. **Computed Tomography Findings of Immune Checkpoint Inhibitor–Induced Interstitial Lung Disease **eFigure 2. **The Proportion of Carcinomas in Total Participants and Immune Checkpoint Inhibitor–Induced Interstitial Lung Disease Cases
