# Supplementary material for: Association of Preexisting Interstitial Lung Abnormalities With Immune Checkpoint Inhibitor–Induced Interstitial Lung Disease Among Patients With Nonlung Cancers
Source: JAMA Netw Open. 2020 Nov 12;3(11):e2022906. doi: 10.1001/jamanetworkopen.2020.22906 (PMC7662135; doi:10.1001/jamanetworkopen.2020.22906)
Supplement: Supplement. — eTable 1. Spearman Rank Correlation Coefficient in the CT Characteristics Among CT Readers eTable 2. Comparison of Characteristics Between Patients With and Without Interstitial Lung Abnormalities eTable 3. Patient and CT Characteristics eTable 4. Proportion of Patients With Immune Checkpoint Inhibitor–Induced Interstitial Lung Disease eTable 5. Comparison of Characteristics Between Patients With and Without Immune Checkpoint Inhibitor–Induced Interstitial Lung Disease eTable 6. Comparison of CT Findings Between Patients With and Without Immune Checkpoint Inhibitor–Induced Interstitial Lung Disease eFigure 1. Computed Tomography Findings of Immune Checkpoint Inhibitor–Induced Interstitial Lung Disease eFigure 2. The Proportion of Carcinomas in Total Participants and Immune Checkpoint Inhibitor–Induced Interstitial Lung Disease Cases [file jamanetwopen-e2022906-s001.pdf]

Supplemental Online Content

Shimoji K, Masuda T, Yamaguchi K, et al. Association of preexisting interstitial lung abnormalities with immune checkpoint inhibitor–induced interstitial lung disease among patients with nonlung cancers. *JAMA Netw Open*. 2020;3(11):e2022906. doi:10.1001/jamanetworkopen.2020.22906

eTable 1. Spearman Rank Correlation Coefficient in the CT Characteristics Among CT Readers
eTable 2. Comparison of Characteristics Between Patients With and Without Interstitial Lung Abnormalities
eTable 3. Patient and CT Characteristics
eTable 4. Proportion of Patients With Immune Checkpoint Inhibitor–Induced Interstitial Lung Disease
eTable 5. Comparison of Characteristics Between Patients With and Without Immune Checkpoint Inhibitor–Induced Interstitial Lung Disease
eTable 6. Comparison of CT Findings Between Patients With and Without Immune Checkpoint Inhibitor–Induced Interstitial Lung Disease
eFigure 1. Computed Tomography Findings of Immune Checkpoint Inhibitor–Induced Interstitial Lung Disease
eFigure 2. The Proportion of Carcinomas in Total Participants and Immune Checkpoint Inhibitor–Induced Interstitial Lung Disease Cases

This supplemental material has been provided by the authors to give readers additional information about their work.

© 2020 Shimoji K et al. *JAMA Network Open*.

eTable 1. Spearman Rank Correlation Coefficient in the CT Characteristics Among CT Readers

**CT characteristics**	r_s	p-value
Interstitial lung abnormalities	0.83	<.0001
Ground glass attenuation	0.88	<.0001
Reticulation	0.82	<.0001
Honeycomb	1.00	<.0001
Emphysema	0.94	<.0001

CT=computed tomography.

© 2020 Shimoji K et al. *JAMA Network Open*.

eTable 2. Comparison of Characteristics Between Patients With and Without Interstitial Lung Abnormalities

**Patient characteristics**	ILA +	ILA -	p-value
	n = 37	n = 162	
Age			
median (range)	70 (51-88)	64 (20-93)	0.001
Sex			
Male	25 (67.5)	108 (66.6)	
Female	12 (32.4)	54 (33.3)	0.91
Smoking status			
Never	14 (37.8)	72 (44.4)	
Current or Former	21 (56.7)	77 (47.5)	0.37
ECOG PS			
0-1	25 (67.5)	127 (78.3)	
≥2	12 (32.4)	35 (21.6)	0.16
Line of ICI therapy			
1	5 (13.5)	29 (17.9)	
≥2	32 (86.4)	133 (82.0)	0.52

ILA=Interstitial lung abnormalities; ECOG PS=Eastern Cooperative Oncology Group Performance Status; ICI=immune checkpoint inhibitor.

© 2020 Shimoji K et al. *JAMA Network Open*.

eTable 3. Patient and CT Characteristics

**Patient characteristics**					
Tumor type	**Head and neck cancer**	**Melanoma**	**Urological cancer**	**Oral cavity cancer**	**Gastrointestinal cancer**
	n=45	n=42	n=42	n=32	n=28
Age, years					
Median (range)	62 (20-84)	70 (32-93)	68 (42-84)	68 (29-82)	67.5 (25-80)
Sex					
Male	33 (73.3)	22 (52.3)	33 (78.5)	18 (56.2)	19 (67.8)
Female	12 (26.6)	20 (47.6)	9 (21.4)	14 (43.7)	9 (32.1)
Smoking history					
Never	16 (35.5)	30 (71.4)	14 (33.3)	13 (40.6)	7 (25.0)
Current or Former	24 (53.3)	11 (26.1)	25 (59.5)	17 (53.1)	17 (60.7)
Unknown	5 (11.1)	1 (2.3)	3 (7.1)	2 (6.2)	4 (14.2)
ECOG PS					
0–1	33 (73.3)	31 (73.8)	35 (83.3)	25 (78.1)	9 (32.1)
≥2	12 (26.6)	11 (26.1)	7 (16.6)	7 (21.8)	19 (67.8)
**CT characteristics**					
Abnormal finding					
+	41 (91.1)	28 (66.6)	38 (90.4)	30 (93.7)	8 (28.5)
-	4 (8.8)	14 (33.3)	4 (9.5)	2 (6.2)	20 (71.4)
Details of abnormal finding					
Pre-existing interstitial lung abnormalities					

© 2020 Shimoji K et al. *JAMA Network Open.*

+	7 (15.5)	7 (16.6)	10 (23.8)	7 (21.8)	5 (17.8)
-	38 (84.4)	35 (83.3)	32 (76.1)	25 (78.1)	23 (82.1)
Type of interstitial lung abnormalities					
Ground glass attenuation					
+	5 (11.1)	4 (9.5)	6 (14.2)	6 (18.7)	3 (10.7)
-	40 (88.8)	38 (90.4)	36 (85.7)	26 (81.2)	25 (89.2)
Reticulation					
+	2 (4.4)	6 (14.2)	5 (11.9)	3 (9.3)	3 (10.7)
-	43 (95.5)	36 (85.7)	37 (88.0)	29 (90.6)	25 (89.2)
Honeycombing					
+	1 (2.2)	2 (4.7)	1 (2.3)	0 (0.0)	1 (3.5)
-	44 (97.7)	40 (95.2)	41 (97.6)	32 (100.0)	27 (96.4)
Traction bronchiectasis					
+	0 (0.0)	0 (0.0)	0 (0.0)	0 (0.0)	0 (0.0)
-	45 (100.0)	42 (100.0)	42 (100.0)	32 (100.0)	28 (100.0)
Emphysema					
+	11 (24.4)	7 (16.6)	7 (16.6)	9 (28.1)	11 (39.2)
-	34 (75.5)	35 (83.3)	35 (83.3)	23 (71.8)	17 (60.7)
Lung metastasis					
+	25 (55.5)	15 (35.7)	22 (52.3)	18 (56.2)	3 (10.7)
-	20 (44.4)	27 (64.2)	20 (47.6)	14 (43.7)	25 (89.2)
Radiation pneumonitis					
+	2 (4.4)	2 (4.7)	0 (0.0)	2 (6.2)	0 (0.0)

© 2020 Shimoji K et al. *JAMA Network Open.*

-	43 (95.5)	40 (95.2)	42 (100.0)	30 (93.7)	28 (100.0)
Consolidation					
+	1 (2.2)	0 (0.0)	2 (4.7)	4 (12.5)	1 (3.5)
-	44 (97.7)	42 (100.0)	40 (95.2)	28 (87.5)	27 (96.4)
Lymphangiosis carcinomatosa					
+	0 (0.0)	0 (0.0)	2 (4.7)	0 (0.0)	1 (3.5)
-	45 (100.0)	42 (100.0)	40 (95.2)	32 (100.0)	27 (96.4)
**Treatment and tumor response**					
ICI					
Nivolumab	45 (100.0)	40 (95.2)	25 (59.5)	32 (100.0)	26 (92.8)
Pembrolizumab	0 (0.0)	2 (4.7)	17 (40.4)	0 (0.0)	2 (7.1)
Line of ICI therapy					
1	3 (6.6)	22 (52.3)	4 (9.5)	3 (9.3)	2 (7.1)
2	17 (37.7)	14 (33.3)	15 (35.7)	13 (40.6)	4 (14.2)
3	14 (31.1)	5 (11.9)	11 (26.1)	12 (37.5)	10 (35.7)
≥4	11 (24.4)	1 (2.3)	12 (28.5)	4 (12.5)	12 (42.8)
The number of cycles of ICIs					
1	5 (11.1)	3 (7.1)	1 (2.3)	1 (3.1)	4 (14.2)
2	0 (0.0)	3 (7.1)	5 (11.9)	6 (18.7)	4 (14.2)
3	1 (2.2)	2 (4.7)	7 (16.6)	3 (9.3)	2 (7.1)
4	6 (13.3)	6 (14.2)	3 (7.1)	3 (9.3)	3 (10.7)
≥5	33 (73.3)	28 (66.6)	26 (61.9)	19 (59.3)	15 (53.5)
Best tumor response					

© 2020 Shimoji K et al. *JAMA Network Open.*

CR	1 (2.2)	0 (0.0)	0 (0.0)	0 (0.0)	0 (0.0)
PR	8 (17.7)	4 (9.5)	9 (21.4)	5 (15.6)	2 (7.1)
SD	12 (26.6)	12 (28.5)	13 (30.9)	11 (34.3)	5 (17.8)
PD	18 (40.0)	19 (45.2)	17 (40.4)	10 (31.2)	16 (57.1)
Unknown	6 (13.3)	7 (16.6)	3 (7.1)	6 (18.7)	5 (17.8)

ECOG PS=Eastern Cooperative Oncology Group performance status; CT=computed tomography; ICI=immune checkpoint inhibitor; CR=complete response; PR=partial response; SD=stable disease; PD=Progressive disease.

© 2020 Shimoji K et al. *JAMA Network Open.*

eTable 4. Proportion of Patients With Immune Checkpoint Inhibitor–Induced Interstitial Lung Disease

**Type of cancer**	ICI-ILD +<br>n (%)	ICI-ILD –<br>n (%)
Head and Neck cancer	5 (11.1)	40 (88.8)
Malignant melanoma	2 (4.7)	40 (95.2)
Urological cancer	4 (9.5)	38 (90.4)
Oral cancer	4 (12.5)	28 (87.5)
Gastrointestinal cancer	3 (10.7)	25 (89.2)

ICI-ILD=Immune checkpoint inhibitor-induced interstitial lung disease

© 2020 Shimoji K et al. *JAMA Network Open*.

eTable 5. Comparison of Characteristics Between Patients With and Without Immune Checkpoint Inhibitor–Induced Interstitial Lung Disease

**Patient characteristics**	ICI-ILD +	ICI-ILD -	p-value
**Head and Neck cancer**	n = 5 (11.1%)	n = 40 (88.8%)	
Age			
median (range)	58 (20-71)	62 (21-84)	0.34
Sex			
Male	4 (80.0)	29 (72.5)	
Female	1 (20.0)	11 (27.5)	0.72
Smoking history			
Never	2 (40.0)	14 (35.0)	
Current or Former	2 (40.0)	22 (55.0)	0.66
ECOG PS			
0-1	4 (80.0)	29 (72.5)	
≥2	1 (20.0)	11 (27.5)	0.72
Line ICI therapy			
1	0 (0.0)	3 (7.5)	
≥2	5 (100.0)	37 (92.5)	0.52
Best tumor response			
CR+PR	2 (40.0)	7 (17.5)	
SD+PD	3 (60.0)	27 (67.5)	0.33
**Malignant melanoma**	n = 2 (4.7%)	n = 40 (95.2%)	
Age			
median (range)	76.5 (74-79)	69 (32-93)	0.42
Sex			
Male	2 (100.0)	20 (50.0)	
Female	0 (0.0)	20 (50.0)	0.16
Smoking history			
Never	2 (100.0)	28 (70.0)	
Current or Former	0 (0.0)	11 (27.5)	0.38
ECOG PS			

© 2020 Shimoji K et al. *JAMA Network Open*.

0-1	1 (50.0)	30 (75.0)	
≥2	1 (50.0)	10 (25.0)	0.43
Line ICI therapy			
1	2 (100.0)	20 (50.0)	
≥2	0 (0.0)	20 (50.0)	0.16
Best tumor response			
CR+PR	0 (0.0)	4 (10.0)	
SD+PD	2 (100.0)	29 (72.5)	0.60
**Urological cancer**	n = 4 (9.5%)	n = 38 (90.4%)	
Age			
median (range)	72.5 (62-76)	67 (42-84)	0.36
Sex			
Male	4 (100.0)	29 (76.3)	
Female	0 (0.0)	9 (23.6)	0.27
Smoking history			
Never	1 (25.0)	13 (34.2)	
Current or Former	2 (50.0)	23 (60.5)	0.92
ECOG PS			
0-1	2 (50.0)	33 (86.8)	
≥2	2 (50.0)	5 (13.1)	0.06
Line ICI therapy			
1	0 (0.0)	4 (10.5)	
≥2	4 (100.0)	34 (89.4)	0.49
Best tumor response			
CR+PR	2 (50.0)	7 (18.4)	
SD+PD	1 (25.0)	29 (76.3)	0.06
**Oral cancer**	n = 4 (12.5%)	n = 28 (87.5%)	
Age			
median (range)	66 (53-80)	68 (29-82)	0.79
Sex			
Male	2 (50.0)	16 (57.1)	

© 2020 Shimoji K et al. *JAMA Network Open*.

Female	2 (50.0)	12 (42.8)	0.78
Smoking history			
Never	3 (75.0)	10 (35.7)	
Current or Former	1 (25.0)	16 (57.1)	0.17
ECOG PS			
0-1	2 (50.0)	23 (82.1)	
≥2	2 (50.0)	5 (17.8)	0.14
Line ICI therapy			
1	1 (25.0)	2 (7.1)	
≥2	3 (75.0)	26 (92.8)	0.25
Best tumor response			
CR+PR	1 (25.0)	4 (14.2)	
SD+PD	3 (75.0)	18 (64.2)	0.75
**Gastrointestinal cancer**	n = 3 (10.7%)	n = 25 (89.2%)	
Age			
median (range)	71 (70-77)	66 (25-80)	0.30
Sex			
Male	1 (33.3)	18 (72.0)	
Female	2 (66.6)	7 (28.0)	0.17
Smoking history			
Never	2 (66.6)	5 (20.0)	
Current or Former	0 (0.0)	17 (68.0)	0.02
ECOG PS			
0-1	3 (100.0)	16 (64.0)	
≥2	0 (0.0)	9 (36.0)	0.20
Line ICI therapy			
1	1 (33.3)	1 (4.0)	
≥2	2 (66.6)	24 (96.0)	0.06
Best tumor response			
CR+PR	1 (33.3)	1 (4.0)	
SD+PD	0 (0.0)	21 (84.0)	<.001

© 2020 Shimoji K et al. *JAMA Network Open*.

ICI-ILD=Immune checkpoint inhibitor-induced interstitial lung disease; ECOG PS=Eastern Cooperative Oncology Group Performance Status; PD-L1=program death-ligand 1: CR=complete response; PR=partial response; SD=stable disease; PD=progressive disease.

© 2020 Shimoji K et al. *JAMA Network Open*.

eTable 6. Comparison of CT Findings Between Patients With and Without Immune Checkpoint Inhibitor–Induced Interstitial Lung Disease

**CT characteristics**	ICI-ILD +	ICI-ILD -	p-value
**Head and neck cancer**	n = 5	n = 40	
Existing abnormal finding			
+	5 (100.0)	36 (90.0)	
-	0 (0.0)	4 (10.0)	0.45
Pre-existing interstitial lung abnormalities			
+	2 (40.0)	5 (12.5)	
-	3 (60.0)	35 (87.5)	0.10
Type of interstitial lung abnormalities			
Ground glass attenuation			
+	2 (40.0)	3 (7.5)	
-	3 (60.0)	37 (92.5)	0.02
Reticulation			
+	0 (0.0)	2 (5.0)	
-	5 (100.0)	38 (95.0)	0.60
Honeycombing			
+	0 (0.0)	1 (2.5)	
-	5 (100.0)	39 (97.5)	0.72
Traction bronchiectasis			
+	0 (0.0)	0 (0.0)	

© 2020 Shimoji K et al. *JAMA Network Open.*

-	5 (100.0)	40 (100.0)	
Emphysema			
+	0 (0.0)	11 (27.5)	
-	5 (100.0)	29 (72.5)	0.17
Lung metastasis			
+	3 (60.0)	22 (55.0)	
-	2 (40.0)	18 (45.0)	0.83
Radiation pneumonitis			
+	1 (20.0)	1 (2.5)	
-	4 (80.0)	39 (97.5)	0.07
Consolidation			
+	0 (0.0)	1 (2.5)	
-	5 (100.0)	39 (97.5)	0.72
Lymphangiosis carcinomatosa			
+	0 (0.0)	0 (0.0)	
-	5 (100.0)	40 (100.0)	
**Malignant melanoma**	ICI-ILD +	ICI-ILD -	p-value
	n = 2	n = 40	
Existing abnormal finding			
+	1 (50.0)	27 (67.5)	
-	1 (50.0)	13 (32.5)	0.60
Pre-existing interstitial lung abnormalities			
+	0 (0.0)	7 (17.5)	

© 2020 Shimoji K et al. *JAMA Network Open.*

-	2 (100.0)	33 (82.5)	0.51
Type of interstitial lung abnormalities			
Ground glass attenuation			
+	0 (0.0)	4 (10.0)	
-	2 (100.0)	36 (90.0)	0.63
Reticulation			
+	0 (0.0)	6 (15.0)	
-	2 (100.0)	34 (85.0)	0.55
Honeycombing			
+	0 (0.0)	2 (5.0)	
-	2 (100.0)	38 (95.0)	0.74
Traction bronchiectasis			
+	0 (0.0)	0 (0.0)	
-	2 (100.0)	40 (100.0)	
Emphysema			
+	1 (50.0)	6 (15.0)	
-	1 (50.0)	34 (85.0)	0.19
Lung metastasis			
+	0 (0.0)	15 (37.5)	
-	2 (100.0)	25 (62.5)	0.28
Radiation pneumonitis			
+	0 (0.0)	2 (5.0)	
-	2 (100.0)	38 (95.0)	0.74

© 2020 Shimoji K et al. *JAMA Network Open.*

Consolidation			
+	0 (0.0)	0 (0.0)	
-	2 (100.0)	40 (100.0)	
Lymphangiosis carcinomatosa			
+	0 (0.0)	0 (0.0)	
-	2 (100.0)	40 (100.0)	
**Urological cancer**	ICI-ILD +	ICI-ILD -	p-value
	n = 4	n = 38	
Existing abnormal finding			
+	4 (100.0)	34 (89.4)	
-	0 (0.0)	4 (10.5)	0.49
Pre-existing interstitial lung abnormalities			
+	4 (100.0)	6 (15.7)	
-	0 (0.0)	32 (84.2)	<.001
Type of interstitial lung abnormalities			
Ground glass attenuation			
+	2 (50.0)	4 (10.5)	
-	2 (50.0)	34 (89.4)	0.03
Reticulation			
+	2 (50.0)	3 (7.8)	
-	2 (50.0)	35 (92.1)	0.01
Honeycombing			
+	0 (0.0)	1 (2.6)	

© 2020 Shimoji K et al. *JAMA Network Open.*

-	4 (100.0)	37 (97.3)	0.74
Traction bronchiectasis			
+	0 (0.0)	0 (0.0)	
-	4 (100.0)	38 (100.0)	
Emphysema			
+	0 (0.0)	7 (18.4)	
-	4 (100.0)	31 (81.5)	0.34
Lung metastasis			
+	1 (25.0)	21 (55.2)	
-	3 (75.0)	17 (44.7)	0.24
Radiation pneumonitis			
+	0 (0.0)	0 (0.0)	
-	4 (100.0)	38 (100.0)	
Consolidation			
+	0 (0.0)	2 (5.2)	
-	4 (100.0)	36 (94.7)	0.63
Lymphangiosis carcinomatosa			
+	0 (0.0)	2 (5.2)	
-	4 (100.0)	36 (94.7)	0.63
**Oral cavity cancer**	ICI-ILD +	ICI-ILD -	p-value
	n = 4	n = 28	
Existing abnormal finding			
+	4 (100.0)	26 (92.8)	

© 2020 Shimoji K et al. *JAMA Network Open.*

-	0 (0.0)	2 (7.1)	0.58
Pre-existing interstitial lung abnormalities			
+	2 (50.0)	5 (17.8)	
-	2 (50.0)	23 (82.1)	0.14
Type of interstitial lung abnormalities			
Ground glass attenuation			
+	1 (25.0)	5 (17.8)	
-	3 (75.0)	23 (82.1)	0.73
Reticulation			
+	2 (50.0)	1 (3.5)	
-	2 (50.0)	27 (96.4)	0.002
Honeycombing			
+	0 (0.0)	0 (0.0)	
-	4 (100.0)	28 (100.0)	
Traction bronchiectasis			
+	0 (0.0)	0 (0.0)	
-	4 (100.0)	28 (100.0)	
Emphysema			
+	0 (0.0)	9 (32.1)	
-	4 (100.0)	19 (67.8)	0.18
Lung metastasis			
+	1 (25.0)	17 (60.7)	
-	3 (75.0)	11 (39.2)	0.17

© 2020 Shimoji K et al. *JAMA Network Open.*

Radiation pneumonitis			
+	0 (0.0)	2 (7.1)	
-	4 (100.0)	26 (92.8)	0.58
Consolidation			
+	0 (0.0)	4 (14.2)	
-	4 (100.0)	24 (85.7)	0.41
Lymphangiosis carcinomatosa			
+	0 (0.0)	0 (0.0)	
-	4 (100.0)	28 (100.0)	
**Gastrointestinal cancer**	ICI-ILD +	ICI-ILD -	p-value
	n = 3	n = 25	
Existing abnormal finding			
+	2 (66.6)	18 (72.0)	
-	1 (33.3)	7 (28.0)	0.84
Pre-existing interstitial lung abnormalities			
+	2 (66.6)	3 (12.0)	
-	1 (33.3)	22 (88.0)	0.01
Type of interstitial lung abnormalities			
Ground glass attenuation			
+	2 (66.6)	1 (4.0)	
-	1 (33.3)	24 (96.0)	<.001
Reticulation			
+	1 (33.3)	2 (8.0)	

© 2020 Shimoji K et al. *JAMA Network Open.*

-	2 (66.6)	23 (92.0)	0.18
Honeycombing			
+	0 (0.0)	1 (4.0)	
-	3 (100.0)	24 (96.0)	0.72
Traction bronchiectasis			
+	0 (0.0)	0 (0.0)	
-	3 (100.0)	25 (100.0)	
Emphysema			
+	0 (0.0)	11 (44.0)	
-	3 (100.0)	14 (56.0)	0.14
Lung metastasis			
+	0 (0.0)	3 (12.0)	
-	3 (100.0)	22 (88.0)	0.52
Radiation pneumonitis			
+	0 (0.0)	0 (0.0)	
-	3 (100.0)	25 (100.0)	
Consolidation			
+	0 (0.0)	1 (4.0)	
-	3 (100.0)	24 (96.0)	0.72
Lymphangiosis carcinomatosa			
+	0 (0.0)	1 (4.0)	
-	3 (100.0)	24 (96.0)	0.72

CT=computed tomography; ICI-ILD=immune checkpoint inhibitor-induced interstitial lung disease

© 2020 Shimoji K et al. *JAMA Network Open.*

eFigure 1. Computed Tomography Findings of Immune Checkpoint Inhibitor–Induced Interstitial Lung Disease

a b c

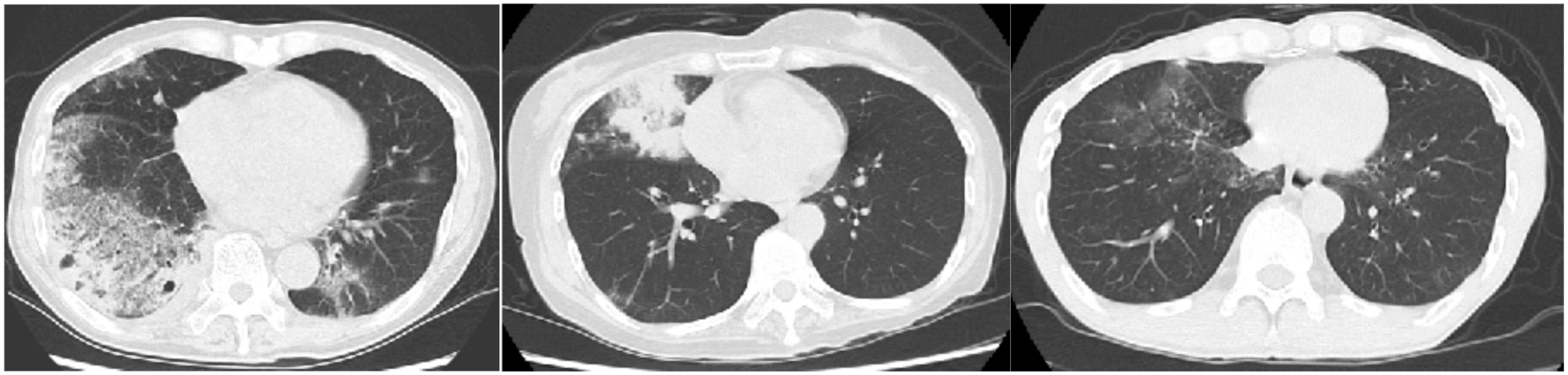

© 2020 Shimoji K et al. *JAMA Network Open.*

eFigure 2. The Proportion of Carcinomas in Total Participants and Immune Checkpoint Inhibitor–Induced Interstitial Lung Disease Cases

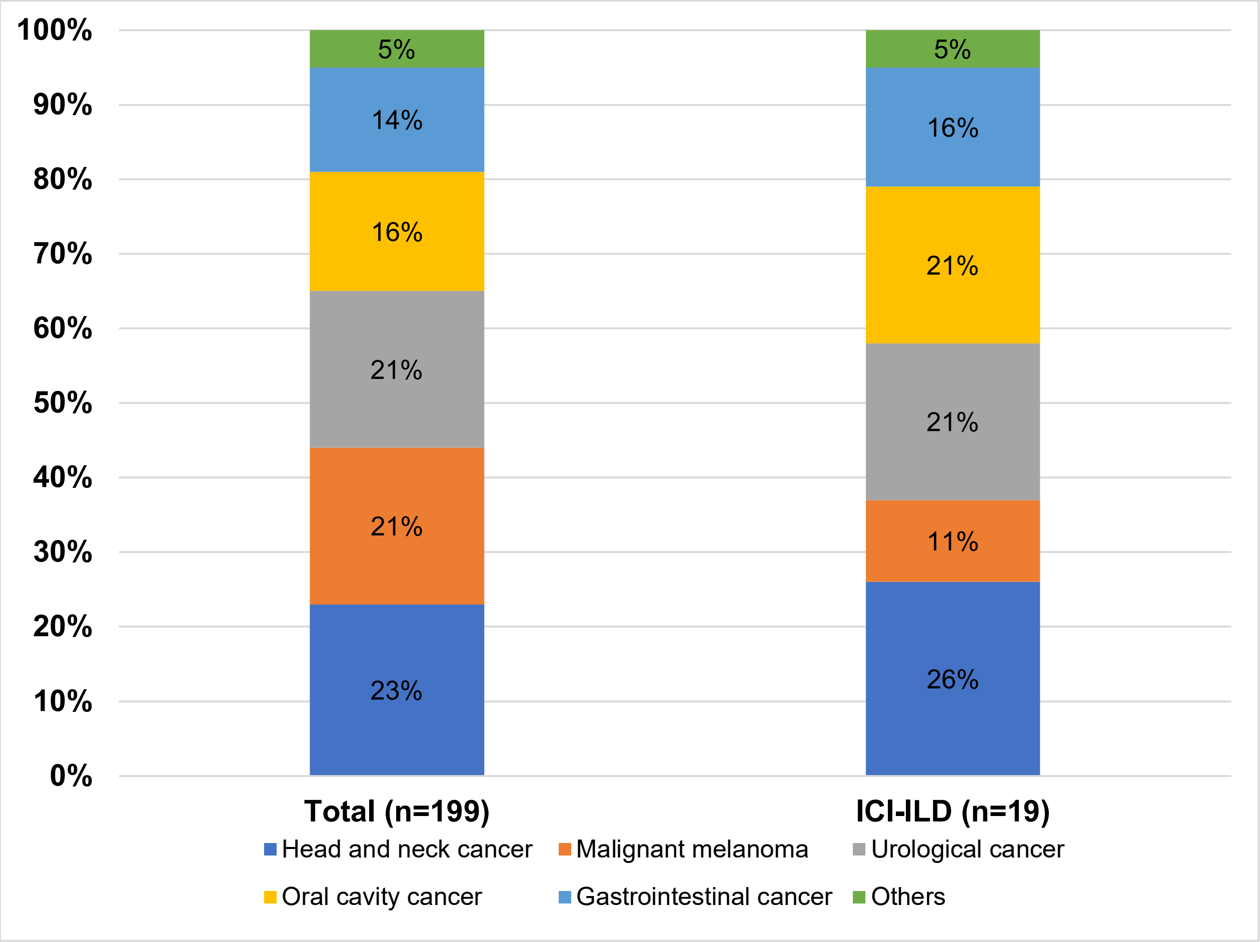

© 2020 Shimoji K et al. *JAMA Network Open.*
